# The sensitivity and specificity of hyperglycosylated hCG (hhCG) levels to reliably diagnose clinical IVF pregnancies at 6 days following embryo transfer

**DOI:** 10.1007/s10815-012-9774-2

**Published:** 2012-04-24

**Authors:** Charles M. Strom, Ruben Bonilla-Guererro, Ke Zhang, Kevin J. Doody, David Tourgeman, Ruben Alvero, Marcelle I. Cedars, Beryl Crossley, Raj Pandian, Rajesh Sharma, Julie Neidich, Denise Salazar

**Affiliations:** 1 Quest Diagnostics Nichols Institute, 33608 Ortega Hiaghway, San Juan Capistrano, CA 92675-2042 USA; 2 Center for Assisted Reproduction, Bedford, TX USA; 3 HRC Fertility, West Los Angeles, CA USA; 4 University of Colorado at Denver, Denver, CO USA; 5 University of California, San Francisco, San Francisco, CA USA

**Keywords:** Pregnancy detection, IVF-ET, Biochemical pregnancy

## Abstract

**Objective:**

To determine the sensitivity and specificity of hyperglycosylated hCG (hhCG) measurements for the diagnosis of clinical pregnancies in the IVF setting and how soon post embryo transfer (ET) a pregnancy can be detected using an ultrasensitive (hhCG) assay. To determine if a single, early hhCG measurement can discriminate between biochemical and clinical pregnancies.

**Design:**

A 4 center prospective blinded clinical trial was performed with patients undergoing IVF-ET. Patients had blood drawn and submitted for hhCG analysis on the day of ET and at days 4, 6, 8, and 12 thereafter. First morning urines were collected and submitted for hhCG analysis on days 0, 4, 6, 8, 10 and 12.

**Setting:**

Fertility Centers

**Outcome Measures:**

Clinical pregnancies were defined as an ultrasound study demonstrating a gestational sac and/or heart beat at appropriate gestational ages.

**Results:**

Fifty-six of 58 enrolled patients completed the study. There were 25 clinical and 6 biochemical pregnancies. For blastocyst transfers, a single serum or urine hhCG measurement identified pregnancies (both biochemical and clinical) at 6 days post ET with 100% sensitivity and specificity. There were 6 biochemical pregnancies, all following blastocyst transfers. All of these pregnancies were identified by lower values.

## Introduction

For couples undergoing in vitro fertilization with embryo transfer (IVF-ET), the interval between embryo transfer and the first pregnancy test can be extremely stressful. One patient describes in agonizing detail the “2WW” or “2 week wait”. Her ordeal: waiting to know if the procedure has been successful [1]. Even when the first pregnancy test reveals a low level of hCG, further tests are usually necessary before a definitive diagnosis of pregnancy can be made. Even after a diagnosis of pregnancy there are several further weeks of anxiety prior to the first ultrasound examination to determine if the pregnancy will fail or succeed.

In 1997 a unique molecule was isolated from the urine of women with invasive trophoblastic disease [2]. Initially called Invasive Trophoblast Antigen (ITA), the name was changed to hyperglycosylated human chorionic gonadotropin (hhCG) when structural analysis revealed that ITA was actually a hyperglycosylated isoform of human chorionic gonadotropin (hCG) sharing an identical 92 amino acid alpha chain and a 145 amino acid beta chain. Both hCG and hhCG are glycoproteins. The 2 isoforms are differentiated by the extent and complexity of sugar residues, with hhCG having more extensive and more complex carbohydrate moieties.

Hyperglycosylated hCG is the primary hCG isoform present during the initial 3 weeks of pregnancy [3–9]. A monoclonal antibody, B152, was raised against hhCG, and has >99 % specificity for hhCG relative to hCG [4, 8, 10]. Of note many antibodies raised to hCG have significant cross reactivity with hhCG [11]. Early in pregnancy, hhCG is the predominant isoform of hCG [12, 13].

Hyperglycosylated hCG is synthesized primarily by cytotrophoblasts, whereas hCG is produced by syncytiotrophoblasts [12, 13]. The function of hhCG in early pregnancy appears to be to facilitate the invasion of the embryo into the uterine wall during implantation [13–15].

There are 3 potential advantages of measuring hhCG rather than hCG for the diagnosis of pregnancy in the IVF-ET setting. Because of the low cross reactivity of the B152 antibody with hCG, there is less interference from the iatrogenic hCG administered during routine IVF to induce final oocyte maturation. Since hhCG is presumably involved in implantation by facilitating trophoblast invasion, hhCG might be an excellent marker for implantation. In addition, a previous study using urinary hhCG measurements, demonstrated that hhCG rose 3 days sooner on average than hCG levels in pregnancies using gestational surrogates. This study also noted the ratio of hhCG to hCG could differentiate between clinical and biochemical pregnancies [5].

We developed an ultrasensitive hhCG assay capable of measuring hhCG at a lower limit of quantitation of 4.580 pg/ml in both urine and serum. We conducted a prospective, blinded, clinical trial of patients undergoing routine IVF in 4 centers in order to determine the sensitivity and specificity of a single hhCG measurement for the detection of pregnancy in the IVF setting and establish how soon after ET an IVF pregnancy could be detected. We also investigated whether hhCG levels could discriminate between clinical and biochemical pregnancies.

## Methods

### Study design

This study was administered by the clinical research organization (CRO) Syteract in San Diego, California and approved by the Western Institutional Review Board and the University of California San Francisco Institutional Review Board.

Four sites participated in the study: The Center for Assisted Reproduction in Bedford TX, HRC Fertility Center in Los Angeles CA, the Advanced Reproductive Medicine Center at University of Colorado in Denver CO, and the University of California San Francisco Center for Reproductive Medicine in San Francisco CA. Contributing centers offered participation to all couples presenting for routine IVF-ET. Couples using donor oocytes or embryos were excluded from the study as were couples using frozen embryos. Participating patients had blood drawn on the day of ET, and at days 4, 6, 8, and 12 thereafter. A first morning urine sample was obtained on days 0, 4, 6, 8, 10, and 12. The samples were identified by case number and transported to the laboratory where analysis was performed within 2 weeks of sample acquisition and data reported to the CRO. The laboratory was blinded. Centers performed routine pregnancy testing and ultrasound confirmations according to their usual protocols.

Each center reported the eventual results for each cycle to the CRO. A clinical pregnancy was defined as a positive pregnancy test using standard practices of the center and an ultrasound demonstrating either a gestational sac or a gestational sac and heart beat at the appropriate gestational age. A biochemical pregnancy was defined as a positive pregnancy test with no gestational sac or heartbeat. A non-pregnancy was defined as a negative pregnancy test. After all data had been obtained, the code was broken and data analyzed. The first patient was enrolled on 11/20/2010 and the final patient was enrolled on 8/29/2011.

### Ultrasensitive hhCG assay

A detailed description of this assay is beyond the scope of this article and will be described elsewhere. Briefly, hhCG is measured using an electrochemiluminescence (ECL) technique performed on a Mesoscale MSD Sector Imager 1250 (Meso Scale Discovery, Gaithersburg, Maryland). Assays are performed in specially constructed 96 well microtiter plates with integrated carbon-ink electrodes at the bottom of each well. Capture antibodies are selectively attached to the electrode surfaces and used in an antibody sandwich assay. ECL-based assays employ compounds that emit light when electrochemically oxidized or reduced under appropriate chemical conditions. The hhCG assay employs two affinity-purified monoclonal antibodies. The hhCG-specific capture antibody B152 is pre-coated onto a specific electrode of the multi-spot well. The signal is generated by the hhCG and hCG reactive B207 antibody conjugated with the MSD sulfo-Tag reagent.

The technical specifications achieved for this assay are an analytic measurement range of 4.580–50,000 pg/ml and an inter-assay variation of 5.39 %, 3.47 %, 5.52 % and 7.68 % for low, mid, mid-high and high level hhCG controls respectively, and an intra-assay variability of 4.51 %, 3.57 %, 5.81 % and 6.51 % for the same controls, respectively. The lower limit of detection is 0.512 pg/ml and the lower limit of quantitation is 4.580 pg/ml.

### Statistical methods

Determination of cut-off values was based on hhCG data distributions with 100 % specificity. R was used as our statistical package [16]. Other comparisons were performed using Chi Square analyses.

A single individual, an M.D., with board certification in Clinical Biochemical Genetics performed and analyzed all test results. All tests were performed between 2 days and 2 weeks of specimen collection. No correction for missing data was made. There were no adverse outcomes.

## Results

There were 58 enrolled patients. Two patients withdrew prior to completing the study leaving 56 completed cycles. Not all women provided samples at all time points. The mean patient age was 34.8 years with a range of 23–44 years. There were 6 Asians, 4 African Americans, 6 Hispanics and 40 Caucasians. A single patient received 5,000 IU of hCG for ovulation induction, another received 6,500 IU while all others were given 10,000 IU. The mean weight of the patients was 71.8 kg with a range of 46 kg–112 kg. Blastocyst transfer was performed in 70 % of cycles and earlier stage embryos transferred in 30 % of cases. The average number of embryos transferred was 1.3 with a range of 1–2. The overall clinical pregnancy rate was with 44 %. There was no significant difference between the pregnancy rate following blastocyst transfers (17 of 39, 44 %) versus earlier stage embryo transfer (8 of 17, 47 %). There were 12 singleton and 13 twin pregnancies. There were no triplets or higher order pregnancies. There were 6 biochemical pregnancies in the series, all of them following blastocyst transfer.

**Fig. 1:**
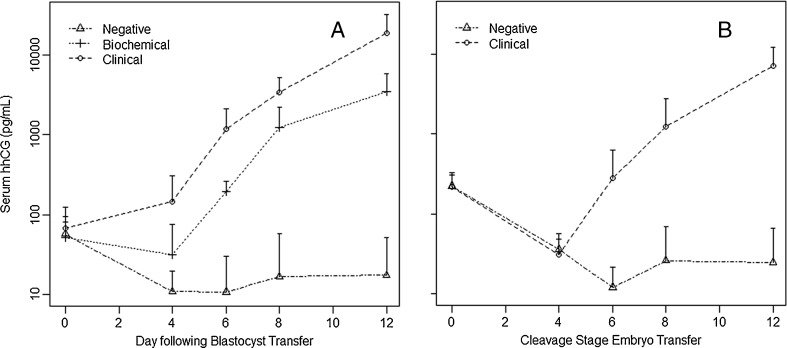
Kinetics of Serum hhCG levels following embryo transfer. **a**: Blastocyst transfer. Diamonds = non-pregnant. Rectangles = Failed pregnancies. Circles = Clinical pregnancies. **b** Cleavage stage embryo transfer. Diamonds = non-pregnant. Circles = Clinical pregnancies

Figure 1a and b represent the kinetics of the rise in hhCG in serum for blastocyst and cleavage stage embryo transfers respectively during the first 12 days following blastocyst transfer. The Y axis is a log scale to accommodate the large increases in hhCG we observed during early pregnancy.

On the day of transfer all but 2 patients had some measureable hhCG, presumably due to the cross-reactivity of the B152 antibody with the exogenously administered hCG. Ten thousand international units is equivalent to 1 g of hCG. With a one percent cross-reactivity of the B152 antibody with hCG, and our assay sensitivity of 4.5 pg/ml, the ability to detect residual hCG in these patients is well within expectation even if >99.9 % of the hCG were already eliminated. By day 6, serum and urine hhCG levels began to rise in clinical pregnancies whereas there was a decline in hhCG levels in non-pregnancies, presumably due to the metabolism of injected hCG for ovulation induction.

Table 1 summarizes the performance of a single serum or urine hhCG value in detecting clinical pregnancies following IVF-ET at various times following ET. For cycles involving blastocyst transfer, a serum hhCG > 75 pg/ml or a urine hhCG > 25 pg/ml has a sensitivity and specificity of 100 % in diagnosing pregnancy. Even as early as day 4, a single serum hhCG value of > 25 pg/ml had an 82 % sensitivity and 87 % specificity.

**Table 1:** Sensitivities and specificities for pregnancy (both clinical and failed) for a single urine or blood hhCG level

		Non-pregnant	Clinical pregnancy	Sensitivity	Specificity
Day 4: Serum hhCG > 25 pg/ml	All	9/24	18/25	72 %	72 %
	Blastocyst	2/15	14/17	82 %	87 %
	Cleavage Stage	7/9	4/8	50 %	22 %
Day 6: Serum hhCG > 75 pg/ml	All	0/19	19/21	90 %	100 %
	Blastocyst	0/10	13/13	100 %	100 %
	Cleavage stage	0/9	6/8	75 %	100 %
Day 8: Serum hhCG > 175 pg/ml	All	0/24	24/25	96 %	100 %
	Blastocyst	0/15	17/17	100 %	100 %
	Cleavage Stage	0/9	7/8	88 %	100 %
Day 12: Serum hhCG > 175 pg/ml	All	23/23	24/24	100 %	100 %
Day 4: Urine hhCG > 15 pg/ml	All	7/24	18/25	71 %	72 %
	Blastocyst	2/15	11/17	65 %	87 %
	Cleavage Stage	5/9	7/8	88 %	44 %
Day 6: Urine hhCG > 25 pg/ml	All	0/24	19/25	76 %	100 %
	Blastocyst	0/15	17/17	100 %	100 %
	Cleavage Stage	0/9	8/2	75 %	100 %
Day 8: Urine hhCG > 25 pg/ml	All	0/24	25/25	100 %	100 %
Day 10, Urine hhCG > 200 pg/ml	All	0/24	25/25	100 %	100 %
Day 12, Urine hhCG > 200 pg/ml	All	0/24	25/25	100 %	100 %

Cycles involving cleavage stage embryo transfer are more variable. There were 2 ultimately clinical pregnancies with delayed hhCG rises until after 8 days. All other clinical pregnancies were detected at days 6 and 8.

As noted above, all patients had a low level of detectable hhCG on the day of transfer. In non-pregnancies, the level of hhCG decreased steadily thereafter. In clinical and biochemical pregnancies the hhCG levels did not fall during the initial 4 days post-ET, indicating probable synthesis of hhCG by the pre-implantation transferred embryos. By day 6, hhCG levels had risen appreciably above day 0 levels. By day 12 hhCG levels were approximately 100 fold higher than day zero levels.

**Fig. 2:**
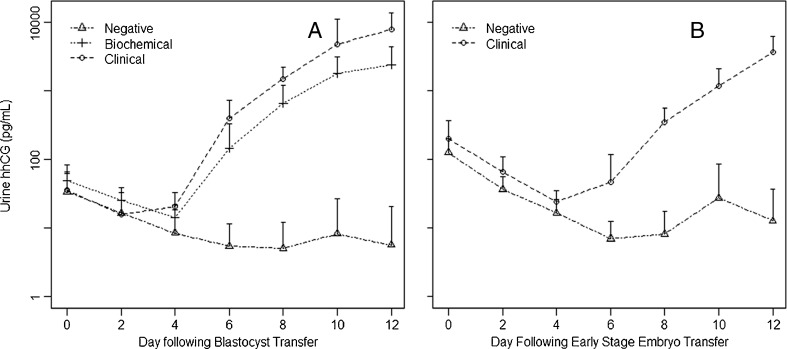
Kinetics of urine hhCG levels following embryo transfer. **a** Blastocyst transfer. Triangles = non-pregnant. Diamonds = Biochemical pregnancies. Circles = Clinical pregnancies. **b** Cleavage stage Embryo Transfer. Triangles: Non-pregnant. Circles: Clinical pregnancies

Figure 2a and b demonstrate the kinetics of urine hhCG values during the post ET period. Although the curves show similar trends, the difference in urine hhCG values between clincal and biochemical pregnancies is not nearly as pronounced as for serum and urine hhCG values cannot discriminate between them.

A commonly used calculation in monitoring early IVF-ET pregnancies is the approximate doubling of hCG levels every 48 h in the period beginning approximately 12 days following ET. We examined our data for the rate of increase for various 48 h time periods for both singleton and twin pregnancies. For the interval between day 4 and day 6, serum hhCG levels increased on average 18 fold in singleton pregnancies and 13 fold in twin pregnancies. The increases were 4 fold for singletons and 5 fold for twins in the interval of day 6 to day 8. Therefore the rate of rise of hhCG is not helpful in differentiating singleton from twin pregnancies. Of note, between days 6 and 8 hhCG rises much more steeply than the approximate doubling of hCG in the second week following ET.

There were 6 failed pregnancies in our series, all following blastocyst transfers. The hhCG levels are lower in biochemical pregnancies than in clinical pregnancies (Figs. 1a and 2a). Table 2 summarizes these data. A serum hhCG level of <300 pg/ml was able to identify all of the failed pregnancies at day 6 and a level of <6,000 pg/ml was able to identify all of the biochemical pregnancies at day 12 (see Table 2). A single clnical pregnancy was below the cut-off value at day 6 and 2 pregnancies were below the cut-off value at day 12. It is important to note that all these data are for blastocyst transfers only. There were no cases of biochemical pregnancies for cleavage stage embryo transfers so no conclusions can be made. Urine hhCG levels did not discriminate between clinical and biochemical pregnancies.

**Table 2:** Discrimination between clinical and failed pregnancies using serum hhCG for blastocyst transfers

Day post ET	Clinical	Chemical	Sensitivity	Specificity
6 day hhCG > = 300 pg/ml	12/13	0/6	100 %	92 %
12 day hhCG> = 6,000 pg/ml	14/16	0/6	100 %	88 %

## Discussion

We performed a blinded prospective trial measuring serum and urine hhCG levels in the 12 days following ET. We determined that following a blastocyst transfer, a single hhCG level measured in urine or serum is sufficient to differentiate pregnant from non-pregnant patients with 100 % accuracy at 6 days post ET. Serum hhCG levels at 6 days and 12 days post ET can distinguish biochemical versus clinical pregnancies with a specificity of 100 % and sensitivities of 92 % and 88 %, at days 6 and 12 respectively. Because of the small numbers in this study, these conclusions must be considered preliminary. Further data will be collected in future series in order to confirm these initial observations.

If used clinically, a patient who has an hhCG level above 300 pg/ml at 6 days following a blastocyst transfer can be told that she probably will have an clinical pregnancy. A patient with a level between 75 pg/ml and 300 pg/ml can be told she likely has a biochemical pregnancy with a small chance it will become a clinical pregnancy. A repeat hhCG level could be drawn at 12 days post ET for these patients. Any patient with an hhCG level below 75 pg/ml can be told she is almost certainly not pregnant. Similarly, at 12 days post blastocyst transfer, a level of > 6,000 pg/ml is indicative of a clinical pregnancy, levels between 175 pg/ml and 6,000 pg/ml indicate a probable biochemical pregnancy, and levels below 175 pg/ml demonstrate a non-pregnancy. It will be up to each individual clinician to interpret the results for his or her patient.

Although a single urine hhCG value at 6 days post blastocyst transfer is able to diagnose a pregnancy, urine values cannot discriminate between biochemical and clinical pregnancies. However, for patients who have difficulty traveling for blood draws, urine hhCG measurement could be a viable option for early pregnancy detection,

Although the data for cleavage stage embryo transfer is not as decisive, hhCG can also be used in these cycles to diagnose pregnancies (both biochemical and clinical) from non-pregnancies in the IVF-ET setting. More data will be necessary to determine the cut-off values to discriminate between biochemical and clinical pregnancies following transfer of cleavage stage embryos.

These data suggest that the primary cause of biochemical pregnancies is deficient implantation as evidenced by low hhCG levels at 6 days following blastocyst transfer. It cannot be determined whether the lower levels of maternal serum hhCG are the cause or the effect of this defective implantation. It is intriguing to speculate that embryos that synthesize less hhCG are less able to invade the uterine wall for a successful implantation. If this is the case, it might be possible to measure hhCG secreted into embryo culture medium in order to select embryos with a higher likelihood of establishing a clinical pregnancy. However, we cannot rule out the possibility that the lower levels of hhCG detected in chemical pregnancies are due to embryos secreting the same amount of hhCG but that less material is transferred to the maternal serum due to faulty implantation.

It should be noted that our sample size is small, containing only 6 biochemical pregnancies. It is certain that not all biochemical pregnancies will eventually be detected by low hhCG levels because there are multiple potential causes of failed pregnancies other than implantation failure including aneuploidy and other genetic abnormalities [17]. Our data would suggest that the most common cause of biochemical pregnancy is implantation failure. Since hhCG is produced by the cytotrophoblasts of the embryo we assume that the implantation failure is do to embryonic and not uterine factors.

Future studies will be necessary to determine if hhCG levels can be used to predict other adverse pregnancy outcomes such as ectopic pregnancies and molar pregnancies. Elevated hhCG levels in the late first trimester (> = 9 week gestation) and second trimester have been shown to be associated with Down Syndrome pregnancies [18–23].

We developed an ultrasensitive hhCG assay for blood and urine. This assay is capable of reliably detecting an IVF-ET pregnancy as early as 6 days post ET in blood and urine and also predict whether a pregnancy will be biochemical or clinical . Although the performance of this testing is best for blastocyst transfers, if one adds 2 days to accommodate for the additional time in tissue culture, pregnancy detection is quite good for cleavage stage embryos. Widespread use of this testing could significantly shorten the period of anxiety sometimes referred to as the “two week wait” for couples undergoing IVF-ET. The ability to discriminate biochemical from clinical pregnancies will alleviate much of the anxiety between the diagnosis of pregnancy and confirmation by ultrasound.
